# Comparative analysis of the medicinal and nutritional components of different varieties of *Pueraria thomsonii* and *Pueraria lobata*

**DOI:** 10.3389/fpls.2023.1115782

**Published:** 2023-03-29

**Authors:** Mei Fu, Mohammad Shah Jahan, Kang Tang, Shizheng Jiang, Juxian Guo, Shanwei Luo, Wenlong Luo, Guihua Li

**Affiliations:** ^1^ Guangdong Key Laboratory for New Technology Research of Vegetables, Vegetable Research Institute, Guangdong Academy of Agricultural Sciences, Guangzhou, China; ^2^ Department of Horticulture, Faculty of Agriculture, Sher-e-Bangla Agricultural University, Dhaka, Bangladesh; ^3^ College of Horticulture, South China Agricultural University, Guangzhou, China

**Keywords:** *Puerariae thomsonii*, *Puerariae lobatae*, flavonoids, starch, dietary fiber

## Abstract

*Pueraria thomsonii* and *Pueraria lobata* are important medicinal plants with unique chemical compositions that are widely used in traditional Chinese medicine. To compare the nutritional and medicinal profiles of these two species, we analyzed the flavonoid, dietary fiber, total starch, and crude protein contents of one *P. lobata* and three *P. thomsonii* varieties using ultra-performance liquid chromatography-tandem mass spectrometry, enzyme weight, acid hydrolysis, and Kjeldahl methods. Furthermore, we used principal component analysis and hierarchical clustering heatmap analysis to separate the data obtained from the *P. thomsonii* and *P. lobata* samples. We detected 279 flavonoid compounds in the two *Pueraria* species, including 90 isoflavones and 78 flavonoids. A large proportion of isoflavones and flavonoids were more abundant in *P. lobata* than in *P. thomsonii.* The total starch content was significantly higher in *P. thomsonii* than in *P. lobata*. By contrast, the soluble dietary fiber, insoluble dietary fiber, and crude protein contents were substantially lower in *P. thomsonii* than in *P. lobata*. Taken together, our results demonstrate that *P. lobata* is better suited for use as a medicine, whereas *P. thomsonii* is better suited as an edible food, and provide a theoretical foundation for developing *P. thomsonii* and *P. lobata* germplasm resources.

## Introduction

*Pueraria*, belonging to the Leguminosae family, is a genus of perennial semi-woody vine that originated in southern, eastern, and southeast Asia. More than 20 species belong to the genus *Pueraria*; among them, *P. thomsonii* and *P. lobata* are economically important food and medicinal plants. *P. thomsonii* contains a higher starch content than *P. lobata* and is known as starch kudzu. *P. lobata* contains abundant isoflavones, particularly puerarin, and is known as kudzu in the Chinese Pharmacopoeia (Wong et al., 2011). At present, most cultivated varieties of *Pueraria* are *P. thomsonii*, while *P. lobata* mostly consists of wild varieties.

Starch in plant-derived foods is the primary source of calories in the human diet. Starch is a type of polysaccharide that is composed of D-glucose monomers. Plants produce starch granules in storage organs such as seeds, aerial and root tubers, and bulbs. Numerous studies have explored the physicochemical characteristics of starch and its modifications (Sangseethong et al., 2005; Liu et al., 2021). *Pueraria* plants produce abundant starch, making them a potential source of starch for use in the food and industrial sectors. *Pueraria* starch can be processed into various cakes, cold drinks, vermicelli, and highly nutritional therapeutic foods. Over the past two decades, numerous *P. thomsonii* cultivars have been developed in China with the wide use of *Pueraria* starch. Dietary fiber is another essential component of the *Pueraria* genus. Used to treat constipation, for detoxification, and to lower blood cholesterol and triglyceride levels (Bazzano, 2008; Trottier et al., 2012; Soliman, 2019; Cronin et al., 2021), dietary fiber is classified based on its solubility. Soluble dietary fiber is soluble in water, can absorb water and expand, and can be digested by microorganisms in the large intestine. By contrast, insoluble dietary fiber does not dissolve in water and cannot be digested in the human gut. Several studies have investigated the nutritional profiles of *P. lobata* and *P. thomsonii* roots (Wong et al., 2015; Shang et al., 2021).

In addition to its nutritional value, *Pueraria* has medicinal properties and is a key component of several traditional Chinese medicines. The root tubers of these plants are rich in bioactive ingredients, particularly flavonoids and isoflavonoids, and their associated derivatives (Wong et al., 2011; Jóźwiak et al., 2018; Wagle et al., 2019). The bioactive components of *Pueraria* have many pharmacological applications. For instance, these components are used to treat cardiovascular and cerebrovascular diseases, hypolipidemia (high cholesterol), hyperglycemia (high blood sugar), and high blood pressure, as well as to inhibit cancer cell activity, expand blood vessels in the brain, and enhance the oxygen supply to the brain (Meezan et al., 2005; Hien et al., 2010; Zhao et al., 2012; Kim et al., 2014; Liu et al., 2015). Therefore, much effort has focused on isolating and identifying the pharmacological constituents of *Pueraria* and analyzing their biosynthetic pathways (Wang et al., 2015; Gao et al., 2016; Wang et al., 2016; Wang et al., 2017; Mocan et al., 2018; Wang et al., 2019). Several common active ingredients, such as daidzin, daidzein, puerarin, and genistin, have been detected in *P. lobata* and *P. thomsonii* (Wong et al., 2011; Wong et al., 2015). Moreover, five isoflavones were detected in *P. lobata* and *P. thomsonii* by near-infrared spectroscopy (NIRS) (Lau et al., 2009). Likewise, 13 isoflavones were identified in both *P. lobata* and *P. thomsonii* using quantitative proton nuclear magnetic resonance (H-NMR) spectrometry (Chen et al., 2014). Although previous studies have offered a glimpse of the active substances present in *P. lobata* and *P. thomsonii*, the research methods employed in these studies are time-consuming and the number of detected active substances is small. Thus, these methods are unlikely to fully reflect the differences between the metabolite complement of *P. lobata* and *P. thomsonii.*

Metabolomics uses cutting-edge analytical techniques to systematically and comprehensively analyze the metabolic contents of organisms (Nicholson and Wilson, 2003). Ultra-performance liquid chromatography-tandem mass spectrometry (UPLC-MS/MS) is commonly used for metabolome studies. In addition to being highly sensitive and highly accurate, this technique offers high resolution and high throughput. UPLC-MS/MS can simultaneously detect numerous metabolites and is widely used in fields such as botany, zoology, and food science (Li et al., 2020; Zou et al., 2020; Li et al., 2021; Zhao et al., 2021). Notably, metabolomics approaches have not been widely used to analyze the pharmacological constituents of these two *Pueraria* species.

To compare the bioactive compositions of these two important *Pueraria* species, we used UPLC-MS/MS to analyze the flavonoid metabolite profiles of three cultivated *P. thomsonii* varieties and one wild *P. lobata* variety. We also analyzed the total starch, dietary fiber, and crude protein contents of these plants. Our results provide a basis for evaluating the medicinal and dietary potential of *P. lobata* and *P. thomsonii* roots and serve as a reference for developing *Pueraria* germplasm resources.

## Materials and methods

### Plant materials

Four *Pueraria* varieties were used as test materials. Three cultivated *P. thomsonii* varieties [Heshui (HS), Fogang (FG), and Guilin (GL)] were collected from the experimental field at Foshan Institute of Agricultural Sciences, Gaoming District, Foshan City, Guangdong Province, China. The *P. lobata* variety [Zhangjiajie (YS)] was collected from Yanghuping Town, Yongding District, Zhangjiajie, Hunan Province, China. All plant materials were harvested in December 2021.

### Sample preparation

The samples were prepared and the metabolites were extracted according to the following steps. Roots were freeze-dried and ground into powder. Approximately 0.1 g of powder was dissolved in 1.2 mL of 70% (v/v) methanol. To ensure that the powder was completely dissolved, samples were vortexed for 30 s every 30 min at least 6 times, and then stored at 4°C overnight. After centrifugation at 12,000 rpm for 10 min, the supernatant was collected and filtered (SCAA-104, 0.22 µm pore size; ANPEL, Shanghai, China) before being used for UPLC-MS/MS analysis.

### UPLC-MS/MS analysis conditions

Flavonoids were profiled on a UPLC-ESI-MS/MS system (UPLC, SHIMADZU Nexera X2) with a C18 column. The two solvents for UPLC consisted of 0.04% (v/v) acetic acid in water (A) and 0.04% (v/v) acetic acid in acetonitrile (B). The gradient program was as follows: (A:B) 95:5 (v/v) at 0 min, 5:95 (v/v) at 11.0 min, 5:95 (v/v) at 12.0 min, 95:5 (v/v) at 12.1 min, and 95:5 (v/v) at 15.0 min; flow rate 0.40 mL/min. A 4-µL sample was injected and the column temperature was maintained at 40°C. For MS, the metabolites were detected on a triple quadrupole–linear ion trap mass spectrometer (API 6500 Q TRAP AB Sciex, CA, USA). The ion source gases were injected at a pressure ranging from 25 to 60 psi. Instrument troubleshooting and mass calibration were performed using 10 µM and 100 µM polypropylene glycol. Multiple reaction monitoring (MRM) mode was used to scan the metabolites. Optimized de-clustering potential (DP) and collision energy (CE) values were used for each MRM transition.

### Determination of Dietary fiber, starch, and crude protein

The crude protein contents were quantified by the Kjeldahl method with slight modifications (Rizvi et al., 2022). Briefly, 200 mg of each sample was ground into fine powder and placed into a 300-mL digestion tube. Five mL sulfuric acid and 2 g accelerator were added to each tube after wetting the sample. Each mixture was incubated at 250 ° for 30 min on a digester. The temperature was raised to 400 ° when H_2_SO_4_ decomposed and emitted while smoke. Each tube was removed from the digester when the solution had turned brownish black. Protein contents were analyzed on an automated protein analyzer.

The enzyme gravimetric method (Nishibata et al., 2009) was used to extract dietary fiber from the samples with slight modifications. Briefly, 100 g of each sample was dried and crushed into fine powder. Each powdered sample was transferred into a high-foot bottle designed to detect dietary fiber. Afterwards, 40 mL of 0.05 ml/L MES-Tris buffer was added and stirred until the sample was completely mixed. The samples were treated with different enzymes, and the treated samples were used to detect the soluble and insoluble dietary fiber.

Total starch content was determined according to the acid hydrolysis method (Rose et al., 1991) with minor modifications. Briefly, 200 mg powdered sample was placed into a 15-mL centrifuge tube to remove the fat and soluble sugars from the sample. Initially, the material was washed several times with a total volume of 150 mL absolute ethanol and 10 mL distilled water. The washed material was transferred into a 50-mL conical flask after removal of the ethanol solution. Subsequently, 3 mL of hydrochloric acid was added to the conical flask and the flask was placed in a boiling water bath for 2 h. Each sample was allowed to cool to room temperature before the pH was adjusted to 7.0. The sample was further incubated for 10 min after adding 2 mL of lead acetate solution, transferred to a 50-mL volumetric flask and diluted with water. Afterwards, the material was filtered and the filtrate was used to quantify total starch contents.

### Data processing

The metabolome analysis was performed by Beijing Biomarker Biotechnology Co., Ltd. Compounds were identified by comparing their ionization spectra with a self-built database. Quantitative analysis was performed using the MRM mode. The data were processed using Analyst 1.6.3 software. Unsupervised principal component analysis (PCA) was performed in R-3.1.1. The results of hierarchical cluster analysis (HCA) of the samples and metabolites are presented as heatmaps. HCA was performed in R using normalized signal intensities of metabolites and displayed as histograms. Similarly, supervised multiple regression orthogonal partial least squares discriminant analysis (OPLS-DA) was performed in R using ropls (R-3.1.1). OPLS-DA modeling was validated with a permutation test (200 permutations). The prediction parameters of the OPLS-DA model included R2X, R2Y, and Q2Y; R2X and R2Y represent the interpretation rate of the built model to the X and Y matrices, respectively, and Q2Y represents the prediction ability of the model. When R2Y and Q2Y parameters are closer to 1, the model is more stable and reliable, and can be used to screen differentially accumulated metabolites (DAMs). Based on the results of OPLS-DA, we analyzed the variable importance in projection (VIP) of the OPLS-DA model from the obtained multivariate analysis. DAMs were determined based on VIP value ≥ 1 and *p*-value < 0.05. The metabolites selected from different comparison groups were presented as volcano plots, plotted in R (R-3.1.1). Each point in the volcano plot represents a metabolite, and the size of the scatter represents the VIP value of the OPLS-DA model. The larger the scatter, the greater the VIP value, and the more reliable the screened differential metabolite is.

## Results

### PCA of flavonoid metabolites in *Pueraria thomsonii* and *Pueraria lobata*

**Figure 1:**
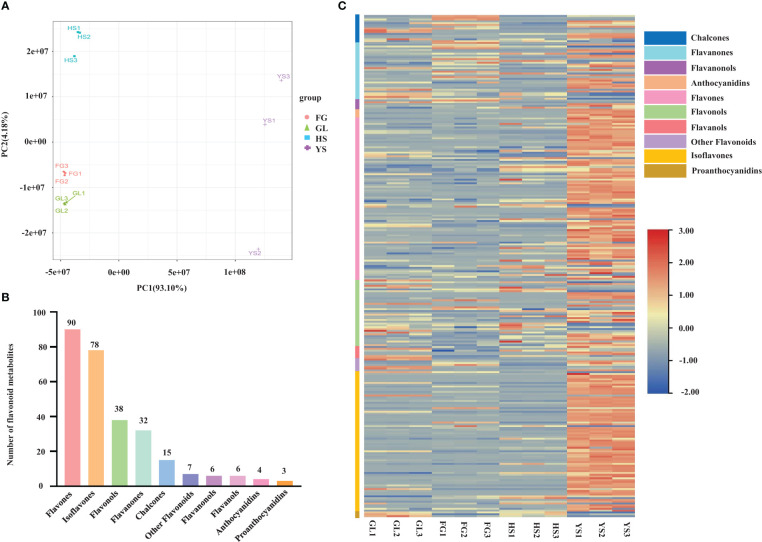
Principal component analysis and metabolite profiles of *P. thomsonii* and *P. lobata*. **(A)** PCA of all samples. **(B)** Classification and quantification of all detected metabolites. **(C)** Heatmap of flavonoid metabolites in *P. thomsonii* and *P. lobata*. HS, *P. thomsonii* variety [Heshui (HS)]; FG, *P. thomsonii* variety [Fogang (FG)]; GL, *P. thomsonii* variety [Guilin (GL)]; YS, *P. lobata* variety [Zhangjiajie (YS)].

We profiled the flavonoid metabolites in one *P. lobata* variety [Zhangjiajie (YS)] and three *P. thomsonii* varieties [Heshui (HS), Fogang (FG), and Guilin (GL)] using UPLC-MS/MS. We performed a PCA to better characterize the overall metabolite differences among the four *Pueraria* varieties and the degree of variability between samples within the same variety. As displayed in the PCA score chart (**Figure 1A**), the contribution by PC1 was 93.10% and that of PC2 was 4.18%. The flavonoid metabolites among the four *Pueraria* varieties were clearly distinguished on the two-dimensional graph, and replicates within the same group clustered, indicating the repeatability and reliability of our data. Moreover, YS was clearly separated from HS, FG, and GL, indicating that *P. thomsonii* and *P. lobata* differ substantially in their metabolite profiles. We identified 279 flavonoid metabolites in the four varieties, consisting of 90 flavonoids, 78 isoflavones, 38 flavonols, 32 dihydroflavones, 15 chalcones, 7 other flavonoids, 6 dihydroflavonols, 6 flavanols, 4 anthocyanins, and 3 proanthocyanidins (**Figure 1B**, **Supplementary Table S1**). The flavonoid metabolites in the four varieties are presented as a heatmap following homogenization (**Figure 1C**). The composition of flavonoid metabolites in YS differed notably from those in HS, FG, and GL, indicating that the flavonoid metabolite compositions of the two species differ substantially.

### OPLS-DA of flavonoid metabolites among the different *Pueraria* varieties

**Figure 2:**
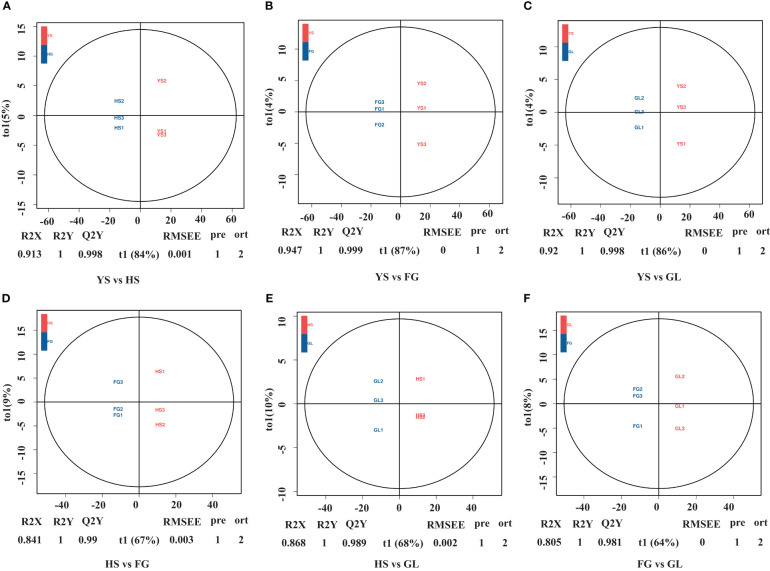
Differential flavonoid accumulation analysis using orthogonal signal correction and orthogonal partial least squares-discriminant analysis. HS, FG, GL, *P. thomsonii* varieties; YS, *P. lobata* variety. Pairwise comparisons YS vs. HS **(A)**, YS vs. FG **(B)**, YS vs. GL **(C)**, HS vs. FG **(D)**, HS vs. GL **(E)**, and FG vs. GL **(F)**.

We used supervised orthogonal signal correction to evaluate the differences in metabolite composition between samples within the same variety, and applied an orthogonal partial least squares-discriminant analysis (OPLS-DA) model to emphasize the distinctions among varieties. OPLS-DA is effective at identifying DAMs as it can be used to optimize population differences. R2X, R2Y, and Q2Y are important parameters for evaluating the OPLS-DA model. Values of R2Y and Q2Y closer to 1 reflect a more stable and reliable model. In addition, Q2Y values above 0.5 are indicative of an effective model, while Q2Y values above 0.9 are indicative of an excellent model. We used the OPLS-DA model to compare the flavonoid metabolite composition of YS vs. HS (R2X=0.913, R2Y=1, Q2Y=0.998; **Figure 2A**), YS vs. FG (R2X=0.947, R2Y=1, Q2Y=0.999; **Figure 2B**), YS vs. GL (R2X=0.920, R2Y=1, Q2Y=0.998; **Figure 2C**), HS vs. FG (R2X=0.841, R2Y=1, Q2Y=0.990; **Figure 2D**), HS vs. GL (R2X=0.868, R2Y=1, Q2Y=0.989; **Figure 2E**), and FG vs. GL (R2X=0.805, R2Y=1, Q2Y=0.981; **Figure 2F**). The high values for R2X, R2Y, and Q2Y in all pairwise comparisons indicate that these analyses were repeatable and reliable and were suitable to screen for DAMs.

### Volcano plot analysis of DAMs among the different *Pueraria* varieties

**Figure 3:**
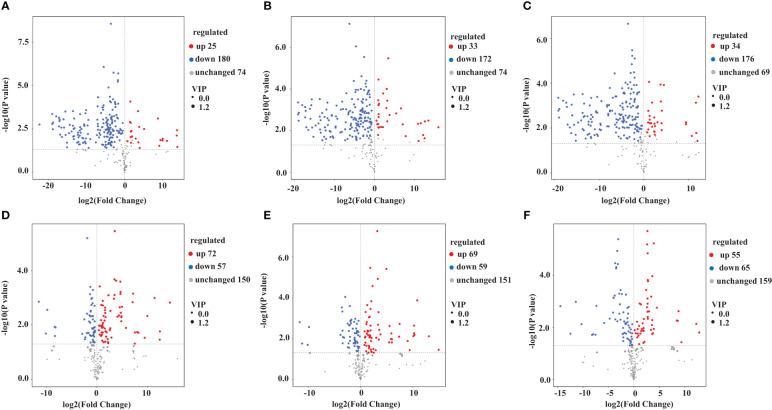
Volcano plot of the differentially accumulated metabolites among the different *Pueraria* varieties. Pairwise comparisons YS vs. HS **(A)**, YS vs. FG **(B)**, YS vs. GL **(C)**, HS vs. FG **(D)**, HS vs. GL **(E)**, and FG vs. GL **(F)**.

We used a VIP value ≥ 1 and *p* < 0.05 as criteria to screen for DAMs. The metabolites that differed between pairs of samples (YS vs. HS, YS vs. FG, YS vs. GL, HS vs. FG, HS vs. GL, and FG vs. GL) are presented as volcano plots in **Figure 3** and the quantification are given in **Supplementary Table S2**. The blue dots in the figure represent down-regulated DAMs, and the red dots represent up-regulated DAMs. We identified 205 significant differentially accumulated flavonoid metabolites between YS and HS, of which 180 were down-regulated in HS compared with YS and 25 were up-regulated in HS compared with YS; 205 between YS and FG (172 down-regulated in FG compared with YS and 33 up-regulated in FG compared with YS); 210 between YS and GL (176 down-regulated in GL compared with YS and 34 up-regulated in GL compared with YS); 129 between HS and FG (57 down-regulated in FG compared with HS and 72 up-regulated in FG compared with HS); 128 between HS and GL (59 down-regulated in GL compared with HS and 69 up-regulated in GL compared with HS); and 120 between FG and GL (65 down-regulated in GL compared with FG and 55 up-regulated in GL compared with FG). These results verify that the complement of flavonoid metabolites differs substantially between YS, HS, FG, and GL.

### Venn diagram and HCA analysis of common DAMs among the different *Pueraria* varieties

**Figure 4:**
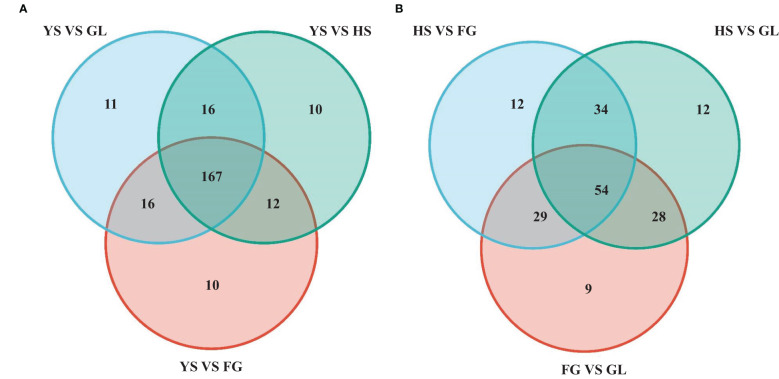
Venn diagram of common differentially accumulated metabolites. HS, FG, GL, *P. thomsonii* varieties; YS, *P. lobata* variety. **(A)** Common differentially accumulated metabolites in comparisons of P. lobata and each P. thomsonii variety. **(B)** Common differentially accumulated metabolites in comparisons of the three P. thomsonii varieties.

**Figure 5:**
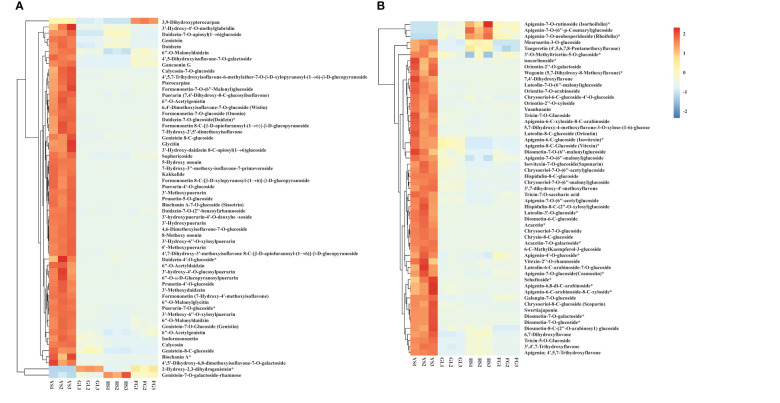
Heatmap of selected differentially accumulated metabolites. **(A)** isoflavones; **(B)** flavones in *P. thomsonii* and *P. lobata*. HS, FG, GL, *P. thomsonii* varieties; YS, *P. lobata* variety. The asterisk indicates that this substance has isomers.

We screened for DAMs that were shared among the different varieties, as shown in the Venn diagram in **Figure 4**. The number of differentially accumulated flavonoid metabolites in YS vs. HS, YS vs. FG, and YS vs. GL was 205, 205, and 210, respectively; however, we identified 167 DAMs in common between each of these comparisons, reflecting the difference between the one *P. lobata* variety (YS) and the three *P. thomsonii* varieties (HS, FG, and GL) (**Figure 4A**). We detected only 54 common DAMs between HS vs. FG, HS vs.GL, and FG vs. GL (**Figure 4B**). These results show that the flavonoid metabolites that underlie the differences between YS vs. HS, YS vs. FG, and YS vs. GL are essentially identical. Furthermore, fewer DAMs were common in comparisons of the three *P. thomsonii* varieties (i.e., 54) than in comparisons of *P. lobata* and each *P. thomsonii* variety (i.e., 167). We next classified the 167 common DAMs into subgroups (**Supplementary Table S3**), of which 59 isoflavones and 55 flavonoids were the major components (**Figure 5**). Compared to *P. thomsonii*, most of the more abundant isoflavones and flavonoids in *P. lobata* were puerarin, daidzein, genistein, biochanin A, acacetin, apigenin, and tricin, and their glycosyl and methyl derivatives. However, the isoflavones and flavonoids mentioned above displayed no clear difference among the three *P. thomsonii* varieties. We further analyzed DAMs among the three *P. thomsonii* varieties. Among these metabolites, 12 metabolites including garbanzol, licoflavonol*, 8-Prenylkaempferol, neobavaisoflavone, and pratensein showed drastic differences in the pairwise comparisons between *P. thomsonii* varieties (**Table 1**), thus defining metabolite signatures that can be used as markers to distinguish the three *P. thomsonii* varieties.

**Table 1:** Differentially accumulated compounds among the three *P. thomsonii* varieties.

Class	Compounds	HS vs. GL		HS vs. FG		FG vs. GL	
		log2FC	VIP	log2FC	VIP	log2FC	VIP
Flavonols	Garbanzol	7.43	1.21	2.90	1.14	4.53	1.25
Flavonols	Licoflavonol*	13.02	1.20	9.47	1.15	3.55	1.23
Flavonols	8-Prenylkaempferol	5.97	1.19	2.58	1.18	3.39	1.22
Isoflavones	Neobavaisoflavone	2.12	1.10	4.99	1.21	-2.87	1.25
Isoflavones	Pratensein	10.19	1.20	7.51	1.11	2.68	1.23
Isoflavones	Glyceollin III	7.35	1.20	9.99	1.21	-2.64	1.24
Chalcones	Licoagrochalcone D*	5.96	1.18	2.17	1.11	3.78	1.21
Other Flavonoids	Licoisoflavanone*	5.88	1.20	2.25	1.11	3.63	1.24
Other Flavonoids	Licoflavone A	2.03	1.10	4.88	1.21	-2.85	1.24
Other Flavonoids	Licoflavone C	3.55	1.19	5.76	1.19	-2.21	1.20
Other Flavonoids	Licoisoflavone A*	5.97	1.19	2.58	1.18	3.39	1.22
Other Flavonoids	(3R)-Vestitol	10.82	1.21	7.19	1.22	3.64	1.25

The asterisk indicates that this substance has isomers.

**Figure 6:**
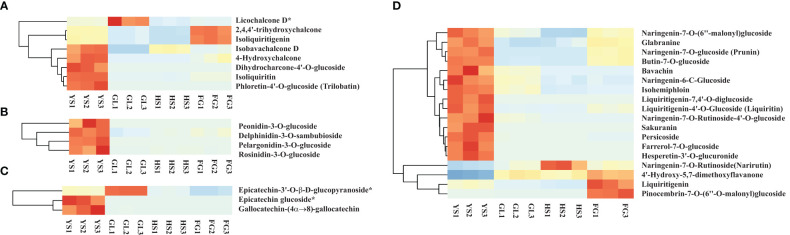
Heatmap of selected differentially accumulated metabolites. **(A)** chalcones; **(B)** anthocyanins; **(C)** flavanols; **(D)** dihydroflavones in *P. thomsonii* and *P. lobata*. HS, FG, GL, *P. thomsonii* varieties; YS, *P. lobata* variety.

**Figure 7:**
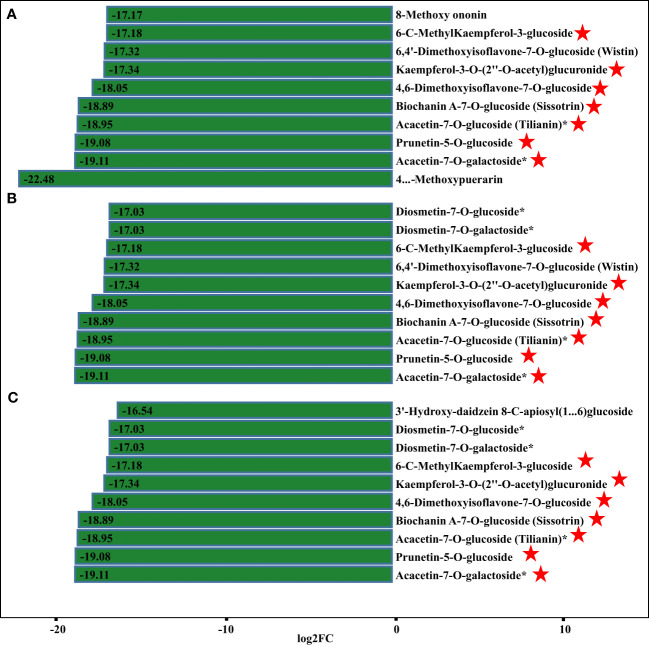
Fold-changes of differentially accumulating metabolites. **(A)** YS vs. HS; **(B)** YS vs. FG; **(C)** YS vs. GL. The pentagram represents the common metabolites of the three comparisons.

Apart from isoflavones and flavonoids, we detected eight chalcones, four anthocyanins, three flavanols, and 18 dihydroflavones that were differentially accumulated in comparisons of *P. lobata* and each *P. thomsonii* variety (**Figure 6**). Among these metabolites, the four anthocyanins peonidin-3-O-glucoside, delphinidin-3-O-sambubioside, pelargonidin-3-O-glucoside, and rosinidin-3-O-glucoside were more abundant in *P. lobata* than in *P. thomsonii* (**Figure 6B**). These results indicate that *P. lobata* accumulated higher levels of flavonoids than *P. thomsonii.* We also detected high amounts of anthocyanin in *P. lobata*. In addition, of the top 10 least abundant DAMs identified in the YS vs. HS, YS vs. FG, and YS vs. GL pairwise comparisons, seven were shared (**Figure 7**): 6-C-methylkaempferol-3-glucoside, kaempferol-3-O-(2’’-O-acetyl) glucuronide, 4,6-dimethoxyisoflavone-7-O-glucoside, biochanin A-7-O-glucoside (Sissotrin), acacetin-7-O-glucoside (tilianin)*, prunetin-5-O-glucoside, and acacetin-7-O-galactoside*. These substances can therefore be used as markers to distinguish between *P. lobata* and *P. thomsonii.*

### Comparative analysis of starch, dietary fiber, and crude protein in *Pueraria thomsonii* and *Pueraria lobate*

In addition to flavonoids, nutrients such as starch, dietary fiber, and crude protein contribute to the nutritional value of *P. thomsonii* and *P. lobata*. We quantified the dietary fiber, total starch, and crude protein contents of *P. thomsonii* and *P. lobata* roots using enzyme gravimetry, acid hydrolysis, and the Kjeldahl method, respectively. The *P. thomsonii* varieties contained more starch than the *P. lobata* variety. We observed the lowest total starch content in YS (449.85 ± 2.61 mg/g sample), and the highest starch content in GL (629.93 ± 3.73 mg/g), followed by FG (618.24 ± 3.54 mg/g) and HS (613.50 ± 1.93 mg/g) (**Table 2**).

**Table 2:** Starch, soluble dietary fiber, insoluble dietary fiber, and crude protein contents in *Puerariae Thomsonii* and *Puerariae Lobatae* varieties tested in this study.

Cultivar name	Starch (mg/g)	Insoluble dietary fiber(g/100g)	soluble dietary fiber(g/100g)	Crude protein (g/kg)
ZhangJiaJie(YS)	449.85 ± 2.61c	10.23 ± 0.28a	3.81 ± 0.30a	106.24 ± 0.70a
HeShui(HS)	613.50 ± 1.93b	4.26 ± 0.14b	0.29 ± 0.05c	72.82 ± 0.91c
FoGang(FG)	618.24 ± 3.54b	3.75 ± 0.35bc	0.17 ± 0.03c	70.95 ± 0.41d
GuiLin(GL)	629.93 ± 3.73a	3.24 ± 0.25c	0.88 ± 0.03b	81.40 ± 0.59b

Different letters indicate significant differences (P<0.05), while the same letter indicates no significant differences.

*P. thomsonii* is known for its low fiber content. Indeed, per 100 g of sample, we measured the highest insoluble dietary fiber content in YS (10.23 ± 0.28 g), followed by HS (4.26 ± 0.14 g), FG (3.75 ± 0.35 g), and GL (3.24 ± 0.25 g). Per 100 g of sample, we detected the highest soluble dietary fiber content in YS (3.81 ± 0.30 g), followed by GL (0.88 ± 0.03 g), HS (0.29 ± 0.05 g), and FG (0.17 ± 0.03 g) (**Table 2**).

The *P. lobata* variety contained the highest crude protein content of the four varieties tested. Per kilogram of sample, the crude protein content was highest in YS (106.24 ± 0.70 g), followed by GL (81.40 ± 0.59 g), HS (72.82 ± 0.91g), and FG (70.95 ± 0.41 g) (**Table 2**). These results demonstrate that the nutritional compositions of *P. thomsonii* and *P. lobata* differ substantially, while those of the three *P. thomsonii* varieties are similar to each other.

## Discussion

*Pueraria* species are found throughout Asia, with China being the leading producer. *Pueraria* is predominantly used in traditional Chinese medicine and as an edible food. The metabolic profiles of *Pueraria* plants have not been extensively investigated. Using UPLC-MS/MS, we identified 279 flavonoid metabolites in *P. lobata* and *P. thomsonii* (**Figure 1B**). PCA and HCA indicated that the metabolite constituents of YL, HS, FG, and GL differ markedly, with YL being clearly distinguishable from HS, FG, and GL (**Figure 1**), supporting the notion that the flavonoid metabolite compositions vary among different *Pueraria* species.

Isoflavones and flavonoids are essential bioactive ingredients of *P. lobata* and *P. thomsonii*. Isoflavones are predominantly found in legumes such as soybean (*Glycine max*) and have essential functions in plant protection and nodule formation (Li et al., 2015). Five isoflavones were identified in the roots of *P. thomsonii* and *P. lobata* plants, including puerarin, formononetin-7-O-glycoside, biochanin A-7-O-glucoside, 7-hydroxy-3”-methoxy-isoflavone-7-primeveroside, and genistein-8-C-apiosyl(1→6)glucoside(Shang et al., 2021). Furthermore, 13 isoflavones were previously detected by H-NMR spectrometry in *P. thomsonii* and *P. lobata*, including puerarin, puerarin 6’’-O-xylopyranoside, daidzin, genistin, and mononetin (Chen et al., 2014). In another study, puerarin, genistin, daidzin, and daidzein were the main ingredients identified in *P. thomsonii* and *P. lobata* samples collected from Australia, China, and the USA (Wong et al., 2015). Here, we determined that 59 isoflavones accumulated to a greater degree in *P. lobata* than in *P. thomsonii* (**Figure 5**). The major isoflavones identified here were puerarin, daidzein, genistein, acacetin, and biochanin A, as well as their glycosylated or methylated derivatives, and these altered metabolites significantly affect the pharmacological activities of isoflavone products (Zhou et al., 2014; Egan, 2020; Wang et al., 2020). Here, we established that puerarin, genistin, and daidzein, and their derivatives, are the major isoflavones in *P. thomsonii* and *P. lobata.* Our results were consistent with studies mentioned above. In addition to the isoflavone metabolites mentioned above, our study revealed isoflavone metabolites that had not been identified in previous studies of *Pueraria* plants. These metabolites can be used as biomarkers to distinguish between *P. lobata* and *P. thomsonii*.

The contents of 52 flavonoids, particularly acacetin, apigenin, tricin, and their derivatives, were higher in *P. lobata* than in *P. thomsonii* (**Figure 5**). These flavonoids possess antiplasmodial, antiperoxodant, anti-inflammatory, and anticancer properties (Cai et al., 2004; Kim et al., 2014; Kim et al., 2017; Wu et al., 2018). Our results are consistent with those of a previous UPLC-MS/MS-based analysis (Shang et al., 2021) that had identified 15 flavonoids, including acacetin-7-O-galactoside, kaempferol-7-O-glucoside, and apigenin-7-O-(6’’-p-Coumaryl) glucoside. Although *P. lobata* and *P. thomsonii* both contain abundant isoflavones and flavonoids, the isoflavone and flavonoid contents were higher in *P. lobata* than in *P. thomsonii*, which may explain why *P. lobata* is commonly used for medicinal purposes. Our findings suggest that variations in the abundance and composition of flavonoids and isoflavones may contribute to the different uses of *P. lobata* and *P. thomsonii* (Chen et al., 2006; Chen et al., 2014; Shang et al., 2021). In addition to the two main bioactive ingredients (isoflavone and flavonoids), four anthocyanins were more abundant in *P. lobata* than in *P. thomsonii* (**Figure 6**). Anthocyanins are water-soluble pigments belonging to the flavonoid class. Anthocyanins not only give plants their color, but also limit the damage caused by biological and abiotic stressors (Abdel-Aal et al., 2006; Gould, 2010; He et al., 2020). Therefore, the high accumulation of anthocyanins in *P. lobata* is consistent with *P. lobata* having a darker color than *P. thomsonii.* Moreover, the anthocyanins mentioned above have not been detected in previous studies focusing on *Pueraria* plants.

*Pueraria* plants have dual applications as medicine and food, and are used to produce both pharmaceutical ingredients and edible starch (Tanner et al., 1979). The starch paste produced by *P. thomsonii* is transparent and difficult to degrade, and the elastic modulus of *P. thomsonii* starch gel is significantly lower than that of potato (*Solanum tuberosum*) starch gel. As a result, *P. thomsonii* starch can be used as a food ingredient as well as a raw industrial material. A previous study had shown that the total starch content of PTR (*Puerariae Thomsonii Radix*) was higher than that of PLR (*Puerariae Lobatae Radix*) (Wong et al., 2015). In this study, the total starch content of *P. thomsonii* roots was higher than that of *P. lobata* roots (**Table 2**); thus, *P. thomsonii* will have high utility in the food industry. Indeed, numerous *P. thomsonii* cultivars with high starch yields have been developed in China for use as food.

Dietary fiber improves the human intestinal microbiota and intestinal peristalsis. We observed that *P. thomsonii* had a lower dietary fiber content than *P. lobata* (**Table 2**). This result was inconsistent with the findings of a previous study (Wong et al., 2015) that established that *P. lobata* roots contained more dietary fiber than *P. thomsonii* roots. This discrepancy may be due to the different varieties used in the two studies. The materials used in the previous study came from three different countries (Australia, China, and the USA), while the materials used in our study all came from China. In addition, the three varieties of *P. thomsonii* used in our study are common cultivated varieties, and have broad market value. Fiber also affects the taste of food. Foods with a high fiber content often lack flavor without processing. For the reasons mentioned above, dietary fiber compounds were found in relatively lower quantities among the three varieties of *P. thomsonii* used in our study. Therefore, *P. thomsonii* with a lower fiber content is useful as food. Crude protein content was higher in *P. lobata* than in *P. thomsonii*. Thus, *P. lobata* has an advantage over *P. thomsonii* in efforts aimed at developing and using crude protein.

## Conclusion

Here, we analyzed the flavonoid profiles of four important *Pueraria* varieties (one *P. lobata* variety and three common cultivated *P. thomsonii* varieties) using UPLC-MS/MS. We identified 279 flavonoid metabolites, of which 167 DAMs were common between YS vs. HS, YS vs. FG, and YS vs. GL. We classified these common metabolites into different subgroups, with 59 isoflavones and 55 flavonoids. A large proportion of isoflavones and flavonoids were more abundant in *P. lobata* than in *P. thomsonii.* The starch content was significantly higher in the *P. thomsonii* varieties than in the *P. lobata* variety, while the cellulose and crude protein contents were significantly lower in *P. thomsonii* varieties than in the *P. lobata* variety. This study provides insight into the differences in medicinal and nutritional profiles between *P. lobata* and *P. thomsonii*, and serves as a theoretical basis for developing *P. lobata* and *P. thomsonii* germplasm resources.

## Data availability statement

The original contributions presented in the study are included in the article/**Supplementary Material**. Further inquiries can be directed to the corresponding author.

## Author contributions

Conceptualization, MF; methodology, KT and SJ; software, WL; resources, JG; writing—original draft preparation, MF and MJ; writing—review and editing, SL; supervision and funding acquisition, GL. All authors contributed to the article and approved the submitted version.

## Supplementary material

Supplementary Table 1 Total flavonoid metabolites in the one *P. lobata* and three *P. thomsonii* varieties tested in this study.

Supplementary Table 2 Differential metabolites among the different *Pueraria* varieties tested in this study.

Supplementary Table 3 List of the 167 common differentially accumulating metabolites in the comparison groups.
